# Editorial: DNA Replication Origins in Microbial Genomes, Volume 2

**DOI:** 10.3389/fmicb.2019.02416

**Published:** 2019-10-23

**Authors:** Feng Gao, Alan C. Leonard

**Affiliations:** ^1^Department of Physics, School of Science, Tianjin University, Tianjin, China; ^2^Frontier Science Center of Synthetic Biology (MOE), Key Laboratory of Systems Bioengineering (MOE), Tianjin University, Tianjin, China; ^3^SynBio Research Platform, Collaborative Innovation Center of Chemical Science and Engineering, Tianjin, China; ^4^Laboratory of Microbial Genetics, Department of Biomedical and Chemical Engineering and Science, Florida Institute of Technology, Melbourne, FL, United States

**Keywords:** bacteria, yeast, replication origin, DNA replication, replication regulation, replication licensing, orisome, replisome

As guest editor, Prof. Gao has organized the Research Topic “DNA Replication Origins in Microbial Genomes” for Frontiers in Microbiology (Gao, 2016). Gratifyingly, the papers published in this volume were highly accessed, and well-received by a wide international audience. Given its previous success we decided to revisit this Research Topic with a second edition in 2017.

We are pleased that this topic remains one of keen interest, and also surprised by the diversity of the manuscripts submitted for the second edition. The field is certainly moving in interesting new directions. We present a total of 11 articles, including 6 original research articles, 4 reviews, and one general commentary, all having undergone rigorous peer review.

Although *Escherichia coli* remains the classic model for studying the mechanisms of DNA replication and regulation in bacteria, there are still uncharted territories and even some surprises. The unique replication origin (*oriC*) encodes instructions for assembly of the initiator protein, DnaA-ATP, into complexes (orisomes) required for the initiation step (Leonard and Méchali, 2013; Wolanski et al., 2015; Katayama et al., 2017), but it remains unclear how orisomes unwind DNA and assist with loading DnaB helicase onto the single-strands. New insights are provided by Sakiyama et al., in this volume, including a model to explain the mechanism of DnaB loading in *E. coli*, and evidence that DnaA AAA+ domain His136 residue directs DnaB to the unwound region. Based on recent studies that show synthetic versions of *oriC* can be activated by the normally inactive DnaA-ADP (Grimwade et al., 2018), Leonard et al. present a new perspective on the requirement for DnaA-ATP in orisome function and timing regulation, and suggest that in *E. coli*, DnaA-ATP is needed for site recognition and occupation instead of mechanical functions. Post-initiation, *E. coli oriC* is sequestered by SeqA protein to prevent re-replication. Surprisingly, Ser36 in the SeqA protein is a target for phosphorylation by the serine-threonine kinase, HipA (Semanjski et al., 2018). However, in this volume, questions about this interesting regulatory feature are raised by Riber et al., who show that mutating the Ser36 residue to alanine (and the loss of phosphorylation) does not affect replication initiation.

*Vibrio cholerae* has emerged as an important model system due to a genome comprising two chromosomes. Many questions are raised about the regulation of origin licensing and once-per cycle replication, as well as chromosome partitioning in multi-chromosome bacteria. These topics are well-represented in this volume. Fournes et al. review once-per cycle regulation of secondary chromosomes with an insightful perspective based on plasmid systems. One of the key checkpoint regulators of *V. cholerae* chromosome II is a region of chromosome I called *crtS* (Baek and Chattoraj, 2014; Val et al., 2016). Based on an *in vivo* screen, Ciaccia et al. show that a global transcription factor, Lrp, binds to *crtS* and plays an important role as a licensing factor for chromosome II. In addition to this crosstalk regulation between transcription and chromosome replication, crosstalk must also exist between bacterial chromosome replication and chromosome partitioning (Marczynski et al.; Taylor et al., 2017). Marczynski et al. review replication-partition crosstalk and discuss how *Vibrio cholerae*, has evolved separate and specific replication and partitioning crosstalk systems for its chromosomes. Important for current and future studies are methods to visualize *oriC* regions, chromosome replication, and partitioning in living bacterial cells (for example, see Ginda et al., 2017; Ramachandran et al., 2018). Here, Trojanowski et al. present an in-depth review on single cell imaging methods.

Unexpectedly, *Vibrio cholera* (NSCV1 and NSCV2) strains were found to contain a single chromosome with two replication origins (Xie et al., 2017), adding another level of intrigue to the two chromosome story. In this volume, Bruhn et al. found that both origins can be active (NSCV1) or one origin can be silenced (NSCV2). It is now clear that multi-origin bacterial chromosomes are more prevalent than anticipated (Gao, 2015; Luo and Gao, 2019; Luo et al., 2019), and some thought-provoking issues of regulation raised by this condition are presented here in a commentary by Das and Chattoraj.

It is clear from the two remaining manuscripts in this volume, that the hunt for replication origins on chromosomes remains a worthwhile effort. Jaworski et al. present the novel structure and function of *oriC* in *Campylobacter jejuni*, the bacterium associated with most foodborne infections worldwide. Eukaryotic microbes must also be included, and Wang and Gao present a comprehensive study of *S. cerevisiae* replication origins from genome-wide and population genomics perspectives.

We hope that readers find these articles both informative and entertaining, and we look forward to an exciting future for replication origin research.

## Author Contributions

FG and AL wrote the manuscript. Both authors read and approved the final manuscript.

### Conflict of Interest

The authors declare that the research was conducted in the absence of any commercial or financial relationships that could be construed as a potential conflict of interest.
